# Arterial ischaemic stroke in adolescents and very young adults—results of a European cohort

**DOI:** 10.1093/esj/aakag003

**Published:** 2026-02-17

**Authors:** Eva Luisa Jung, Manoëlle Kossorotoff, Merel Sanne Ekker, Anna-Lisa Oechsle, Kim Tran Dong, Martin Olivieri, Victoria Lieftüchter, Florian Heinen, Christian Denier, Frank-Erik de Leeuw, Lucia Gerstl

**Affiliations:** Division of Pediatric Neurology and Developmental Medicine, Department of Pediatrics, Dr. v. Hauner Children’s Hospital, University Hospital, LMU Munich, Munich, Germany; Department of Neuropediatrics and Muscle Disorders, Faculty of Medicine, Medical Center, University of Freiburg, Freiburg, Germany; French Center for Pediatric Stroke, Pediatric Neurology Department, APHP University Hospital Necker-Enfants malades, Paris, France; Inserm U1266, Paris, France – Institut de Psychiatrie et Neurosciences de Paris (IPNP), Paris, France; Department of Neurology, Radboud University Nijmegen Medical Center, Nijmegen, The Netherlands; Richterswill, Switzerland; French Center for Pediatric Stroke, Pediatric Neurology Department, APHP University Hospital Necker-Enfants malades, Paris, France; Pediatric Thrombosis and Haemostasis Unit, Dr. von Hauner Children's Hospital, University Hospital, LMU Munich, Munich, Germany; Pediatric Intensive Care Medicine, Dr. von Hauner Children’s Hospital, University Hospital, LMU Munich, Munich, Germany; Division of Pediatric Neurology and Developmental Medicine, Department of Pediatrics, Munich University Center for Children with Medical and Developmental Complexity - iSPZ Hauner MUC, Dr. v. Hauner Children's Hospital, University Hospital, LMU Munich, Munich, Germany; Stroke Units and Department of Neurology of Hôpital Bicêtre, Assistance Publique–Hôpitaux de Paris, Paris Saclay University, Le Kremlin-Bicêtre, France; Department of Neurology, Radboud University Nijmegen Medical Center, Nijmegen, The Netherlands; Division of Pediatric Neurology and Developmental Medicine, Department of Pediatrics, Munich University Center for Children with Medical and Developmental Complexity - iSPZ Hauner MUC, Dr. v. Hauner Children's Hospital, University Hospital, LMU Munich, Munich, Germany

**Keywords:** arterial ischaemic stroke, adolescents, very young adults, risk factors, classification system

## Abstract

**Introduction:**

Arterial ischaemic stroke (AIS) in adolescents and young adults differs from older populations due to its variety of risk factors and aetiologies. This study compares the risk factor profiles and age- and sex-related differences between adolescents and very young adults.

**Patients and methods:**

Pooled data from three multicentre cohort studies in Europe (Germany, France, The Netherlands) were analysed. First-ever symptomatic AIS cases in adolescents (10–18 years) and very young adults (18–30 years) were classified using both a paediatric AIS classification (multiple risk factor category system) and the modified Trial of ORG 10172 in Acute Stroke Treatment criteria for adults.

**Results:**

The cohort includes 142 adolescents (median age 15) and 131 very young adults (median age 25). Using the adult AIS classification, cryptogenic stroke was most common (43% in adolescents, 32% in young adults). Thirty-two percent had other determined aetiologies. The paediatric classification showed similar results regarding identified causes; 29% resulted in cryptogenic stroke, respectively. Sex distribution differed by age: male predominance in adolescents (61%) and female predominance in young adults (57%).

**Discussion:**

Adolescence and young adulthood may represent a shared age group with similar causes and risk profiles for AIS. The high rate of cryptogenic stroke suggests potential limitations in current classification systems for AIS aetiology at these ages.

**Conclusion:**

This cohort highlights the need for an age-adapted risk factor system that accounts for the transitional nature and the complexity of stroke in this age group.

## Graphical Abstract

**Graphical Abstract:**
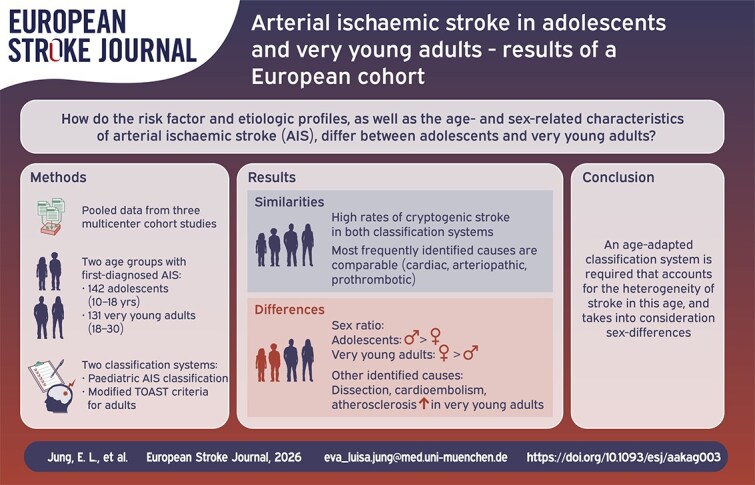

## Introduction

While arterial ischaemic stroke (AIS) in young adults (<50 years) or in paediatric patients have been well described, studies addressing specifically AIS in adolescents or in early adult age (<30 years) are scarce. Adolescence and very young adulthood represent a transition period between early childhood and full adulthood harbouring unique features in physiology and health issues differing from young children and adults. This unique physiological and complex developmental period of this life span is notably characterized by hormonal changes, growth and increased exposure to exogenous factors such as tobacco, alcohol, drugs or contraceptives. Consequently, AIS in adolescents and very young adults is very heterogenous and differs from stroke in later adulthood due to its wide variety of risk factors and aetiologies.

Stroke is well known to be associated with high morbidity and mortality. When it occurs at young age, the impact on life can be tremendous resulting in significant and persisting physical, cognitive and social impairments.^1–6^ Socioeconomic costs due to persisting brain damage after stroke in young are high. AIS in this period of life deserves better understanding of its specific causes. First, because adolescence is one of the few peak periods^7–10^ of paediatric AIS incidence, with an annual incidence of 0.54 to 2.4 per 100,000.^11,12^ Secondly, because longitudinal studies showed an increase of AIS stroke incidence in young adults over time, unlike most other diseases.^13–17^ Thirdly, because the exact pathophysiological mechanisms resulting in AIS in that age are not clarified yet. Furthermore, concerning strokes in young adults (18–49 years) up to one-third are classified as cryptogenic with common classification systems such as the TOAST (Trial of ORG 10172 in Acute Stroke Treatment) classification, usually reflecting the risk factor profile of older age groups, often > 65 years.^18,19^ It has been shown that in the major group of young adults with cryptogenic stroke more risk factors can be found when using a paediatric risk factor classification in which heterogeneity of stroke aetiology in the young becomes evident, providing insight into new causes and mechanisms for strokes in young adults.^20^

Until now only limited data exist about characteristics of AIS during this transitional life stage. This analysis aims to provide an overview of the variations and similarities in the risk factor profile of adolescents (10–18 years) and very young adults (18–30 years) with special attention to age- and sex-related differences and the variety of single and multiple risk factors.

## Patients and methods

### Data availability/patients and study design

Pooled data from 3 different multicentre cohort studies are assembled to address AIS in adolescents (10–18 years) and very young adults (18–30 years). All studies were recently conducted in Northwestern Europe (Germany, France, Netherlands) between 2007 and 2021.

Patients were diagnosed with first-ever symptomatic AIS defined as a focal neurologic deficit with acute onset and radiological evidence of recent brain infarction on magnetic resonance imaging. Further local inclusion criteria included screening according to the ICD-10 (cerebral infarction: code I63, with the exclusion of code I63.6, cerebral venous thrombosis),^12^ diffusion weighted imaging positive lesions (DWI+) on magnetic resonance imaging in patients with transient symptoms (<24 h)^21^ and checks for plausibility by paediatric neurologist and specialist for paediatric haemostaseology.^9^

Adolescence was defined according to the World Health Organization and American Psychological Association (APA) with the age cutoff of 10–18 years.^22,23^ Definition of young adulthood is in contrast slightly varying depending on the context, generally describing the late teens through the twenties. In this study, cut-off age of 30 years was used for very young adults, also based on the predefined age groups of the Dutch ODYSSEY study.^21^

For this data analysis we included adolescents (10–18 years) from German ESPED (Erhebungseinheit für Seltene Pädiatrische Erkrankungen in Deutschland) childhood stroke study and from a French retrospective cohort study. The German study is a prospective epidemiological study conducted via ESPED, a hospital-based German nation-wide surveillance unit for rare paediatric diseases.^9^ Children aged 28 days to 18 years with first AIS between January 2015 and December 2017 were included. 164 children in total were reported. It focused on the investigation of incidence, age-dependent clinical presentation, risk factors and the medical care situation in Germany.^9^ For the present study 82 adolescents (10–18 years) were included.

The French retrospective cohort study evaluated all consecutive patients aged 10–18 years with the diagnosis of a first-ever ischaemic stroke hospitalized between 2007 and 2017 in 10 French academic centres, including 60 patients.^12^ The aim of this study was to describe the clinical and neuroradiologic features, aetiologies, initial management and outcome of ischaemic stroke in adolescents.

The group of very young adults (18–30 years) is formed from data of the Dutch Observational Dutch Young Symptomatic StrokE studY (ODYSSEY) study. The ODYSSEY is a multicentre prospective cohort study conducted in 17 hospitals in the Netherlands between 2013 and 2021, consisting in 1322 patients aged 18–49 years with a first-ever TIA, ischaemic stroke or intracerebral hemorrhage.^21^ It was designed to prospectively investigate the aetiology and prognosis after a young stroke. For the present study, 131 patients aged 18–30 years were included.

### Data collection

Data from all three studies were originally collected independently using standardized medical record review procedures. For the present analysis, the principal investigators jointly reviewed and harmonized the underlying definitions of the already collected variables to ensure consistency across studies. Variables that were collected in one study but not in another were not eligible for inclusion in the pooled analysis. Consequently, the current analysis is based on a restricted, harmonized dataset comprising only variables that were consistently available across all three cohorts. Variables with no recorded information for a given participant were assumed to be absent for that individual.

### Statistical analyses

For this analysis we used pooled data for diagnosed AIS at the age of ≥10 to <18 years (group of adolescents) and at the age of ≥18 to ≤30 years (group of very young adults).

We reported categorical variables as absolute numbers and percentage of the available population unless otherwise stated. Continuous variables were reported as median with the interquartile range (IQR). Age- and sex-specific analyses for causes and risk factors were performed by the 2 age-groups. For comparison, χ^2^ testing or Fisher’s exact test were applied. Binomial test was used for comparing sex distributions. The significance level for all analyses was 5%. All statistics have been calculated using SAS, version 9.4 (SAS Institute Inc., Cary, NC, USA).

### Risk factor and causes of stroke

Risk factors and the cause of AIS were systematically assessed for all patients. They were classified into the following nine different categories, standing for a multiple risk factor category system which has been used before in the German ESPED study to classify paediatric stroke: Cardiac disorders, Prothrombotic risk factor, Arteriopathy, Acute systemic disorders, Haemato-oncological disorders, Metabolic disorders, Chronic head and neck disorders, Other disorders and No risk factor found.^9^  Table 1 summarizes the risk factors in detail and their prevalence among the age groups.

**Table 1:** Stroke aetiology according to the paediatric approach of risk factor categorization, stratified by age and sex.

**Risk factor category**		**Adolescents (*n* = 142)**	**Very young adults (*n* = 131)**
**Cardiac**	Arrythmia Congenital/acquired heart disease Patent foramen ovale Endocarditis Cardiac surgery or catheterization Other	1 9 19 3 1 7	2 2 29 - 1 3
**Prothrombotic**	Protein C deficiency Protein S deficiency Factor V Leiden (G1691A) Prothrombin mutation (G20210A) MTHFR (C677T) Hyperlipoproteinemia Increased factor VIII Other	3 5 5 - 3 6 6 4	- 1 5 1 - - 5 12
**Arteriopathy**	Focal cerebral arteriopathy Para-/postinfectious vasculitis Moyamoya disease Arterial dissection Fibromuscular dysplasia Systemic lupus erythematosus Aneurysm Primary CNS vasculitis Other	4 3 2 10 - 2 1 - 13	- 1 1 19 - 1 - - 1
**Acute systemic**	Infectious disease or fever/flue like symptoms before stroke	10	12
**Haemato-oncological**	Sickle cell disease Haemolytic anaemia Iron deficiency anaemia Other	2 1 - 8	- - - -
**Metabolic**	Mitochondrial disease Fabry disease Homocystinuria CDG syndrome Other	2 - - - -	- - 3 - 2
**Chronic head and neck disorders**	Migraine	14	45
**Others**	Connective tissue disorders Ehlers-Danlos syndrome Marfan syndrome Other Genetic disorders Trauma	- - 1 1 11	1 1 - 1 4

Descriptive data summarizing the risk factors.

Furthermore, pooled data for AIS in both age groups were classified according to the modified TOAST criteria.^24^ The classification system divides ischaemic strokes into five main categories based on their likely aetiology in order to support diagnosis and treatment: (1) Large artery atherosclerosis: Strokes due to significant atherosclerotic plaque in major arteries. (2) Cardioembolism: Strokes caused by emboli from cardiac sources. (3) Small vessel occlusion: Strokes from small arterial blockage. (4) Stroke of other determined aetiology: Strokes with rare causes like vasculitis. (5) Stroke of undetermined aetiology (cryptogenic).

### Ethical approvals

The different cohort studies were conducted with the approval of their local Ethics Committee.^9,12,21^ Ethical approval of the German study was obtained from the Ethics Committee of the Medical Faculty of the Ludwig-Maximilians-University, Munich, No. 42-15 (5 April 2015).

The French study was conducted according to French ethics laws with the approval of the local Institutional Review Board/Independent Ethics Committee, Comité de Protection des Personnes CPP VII. The Dutch study was approved by the Medical Review Ethics Committee region Arnhem-Nijmegen (NL41531.091.12). Retrospective combined data analysis in this study is based only on anonymized data and therefore did not require new ethics approval.

## Results

### Demographics

**Figure 1:**
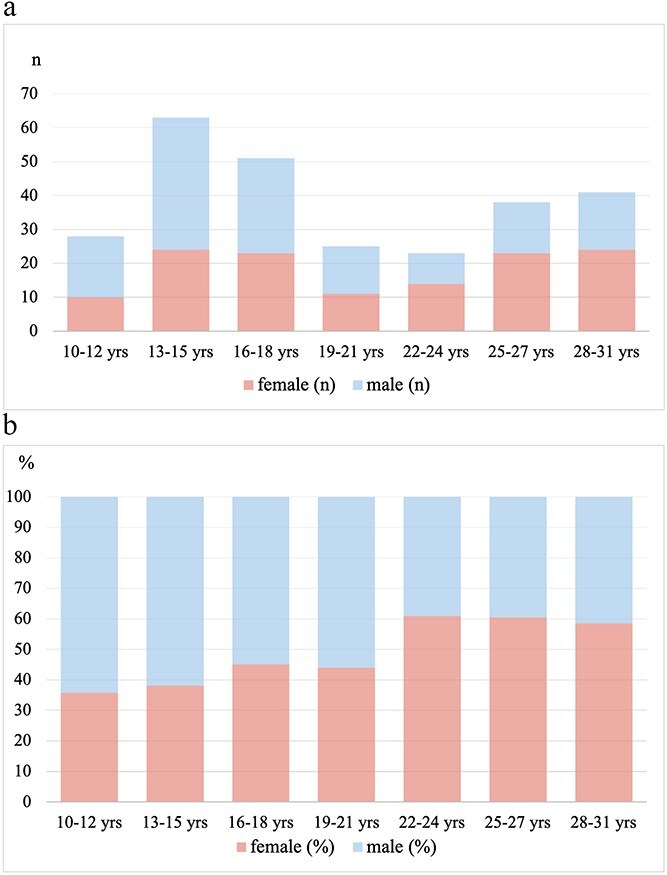
Gender distribution in age categories, presented in (a) number of participants (b) percent of frequency (upper section of each bar: male, lower section of each bar: female).

Pooling the data from the three studies, 142 adolescents (82 from the German study and 60 from the French study) and 131 very young adults (from the Dutch study) with a first-diagnosed AIS were included. Median age of adolescents was 15 years (IQR 13–16) and 25 years (IQR 22–28) in very young adults. Median age of the whole group was 17 years (IQR 15–25) (Table 2). There were significantly more affected males (61%) than females (39%, *P* = .01) in the adolescent’s group, whereas in very young adults sex ratio shifted towards more females (57%) compared to males (43%, *P* = .097) (Figure 1).

**Table 2:** Baseline demographic characteristics of the complete cohort and age-defined subgroups (adolescents and very young adults).

**Age group**	***n***	**Median age, years (IQR)**	**Sex, % female: male**
**Complete cohort**	273	17 (15–25)	48: 52
**Adolescents**	142	15 (13–16)	39: 61^a^
**Very young adults**	131	25 (22–28)	57: 43

^a^ *P*-value <.05 was considered statistically significant.

### Stroke aetiology according to the paediatric AIS classification

Using the above-mentioned paediatric classification, cryptogenic stroke with no identified risk factor was the most frequent category, with a similar rate in both age groups (29%). The three most frequently identified causes and risk factors of AIS were similar between adolescents and very young adults: cardiac disorders (26% and 27%, respectively, not significant), arteriopathies (24% and 18%, respectively, not significant), and prothrombotic risk factors (18% and 18%, respectively, not significant). The incidence of haemato-oncological risk factors was higher in adolescents than in very young adults (*n* = 11, 8%, and *n* = 0, respectively, *P* = .0009), whereas migraine was more frequent in very young adults than in adolescents (*n* = 45, 34%, and *n* = 14, 10%, respectively, *P* < .0001) (Table 3).

**Table 3:** Prevalence of risk factors by age according to the paediatric approach of risk factor categorization (percentages add up to >100% as a relevant number of patients has more than one risk factor category).

**Risk factor category**	**Adolescents (*n* = 142)**	**Very young adults (*n* = 131)**	***P*-value** ^**a**^
**Cardiac**	37 (26%)	36 (27%)	.79
**Prothrombotic**	26 (18%)	24 (18%)	1.00
**Arteriopathy**	34 (24%)	23 (18%)	.19
**Acute systemic**	10 (7%)	12 (9%)	.52
**Haemato-oncological**	11 (8%)	0 (0%)	**.0009**
**Metabolic**	2 (1%)	5 (4%)	.27
**Migraine**	14 (10%)	45 (34%)	**<.0001**
**Others** ^b^	13 (9%)	7 (5%)	.23
**No risk factors**	41 (29%)	38 (29%)	.98

^a^ *P*-values are obtained from χ^2^ or Fisher’s exact tests, *P*-values <.05 were considered to be statistically significant and printed in bold.

^b^Connective tissue disorders, genetic disorders, trauma.

Sex stratification showed a non-significant tendency for a female predominance of prothrombotic risk factors in very young adults (*n* = 18, 24%, and *n* = 6, 11%, respectively, *P* = .05). In adolescents, migraine was more frequent in females than males (17% and 6%, respectively, *P* = .04).

### Focus on arterial dissection

Whereas global arteriopathy rates are comparable between the two age groups, incidence for arterial dissection (AD) is higher in very young adults, representing the predominate type of arteriopathy in very young adults (82.6%, *n* = 19/23), but not in adolescents (29.4%, *n* = 10/34). In total, 14.5% (*n* = 19) of very young adults were affected by AD compared to 7% (*n* = 10) of adolescents (*P* = .0456), without significant sex-related difference in any age group.

### Stroke aetiology according to adult AIS classification

Using the TOAST classification, cryptogenic stroke with undetermined stroke aetiology was the most frequent category in both groups, affecting 50 adolescents (43%) and 42 very young adults (32%, *P* = .10). With this classification, the most frequently identified cause of AIS could not be caught in a definite category, as 32% of patients in each age group were classified as “other determined etiology” for AIS. Determined AIS causes corresponding to a definite category were different between adolescents and very young adults, with cardioembolism higher in very young adults than in adolescents (*n* = 35, 27% and *n* = 23, 17%, respectively, *P* = .03). Some causes were rare in very young adults and exceptional in adolescents (*n* = 6, 5%, and *n* = 0, respectively for large-artery sclerosis, *P* = .01; *n* = 4, 3% and *n* = 0, respectively for small vessel occlusion). Multiple causes for stroke were more frequent in adolescents than in very young adults (*n* = 12, 9% and *n* = 2, 2%, respectively, *P* = .01). In both age groups, no sex-related difference was observed between the main categories of the TOAST classification system (Table 4).

**Table 4:** Categorization by age according to TOAST (only a single primary cause was assigned according to the TOAST definition).

**Toast**	**Adolescents (*n* = 138)**	**Very young adults (*n* = 131)**	***P*-value** ^**a**^
**Large-artery atherosclerosis**	-	6 (5%)	**.01**
**Cardioembolism**	23 (17%)	35 (27%)	**.03**
**Small-vessel occlusion**	-	4 (3%)	.05
**Other determined aetiology**	44 (32%)	42 (32%)	.85
**Undetermined aetiology (cryptogenic)**	59 (43%)	42 (32%)	.10
**Multiple causes**	12 (9%)	2 (2%)	**.01**

^a^ *P*-values are obtained from χ^2^ or Fisher’s exact tests, *P*-values <.05 were considered to be statistically significant and printed in bold.

## Discussion

This study analysing data from 142 adolescents and 131 very young adults from three European countries with a first ever AIS demonstrated strong similarities between the 2 age groups, suggesting they may represent a unique age group considering AIS cause and risk factors. The high rate of cryptogenic stroke in both adolescents and very young adult groups also raises issues about the accuracy of stroke cause inquiry and of the classification used to categorize AIS aetiology.

Adolescents and very young adult groups were different from the global adults group. The TOAST classification was designed to categorize AIS cause in adults. Our study showed that this supposedly adequate classification for adult stroke did not correctly describe stroke cause in very young adults, with 32% of them considered cryptogenic. In contrast, this result was close to what was observed in adolescents (43% cryptogenic). Respectively, another 32% count into the category of other determined causes, without providing specific details on aetiology and pathogenesis. No large vessel atheromatous disease or small vessel disease was reported in this cohort, contrary to what is reported in young adult studies.^25,26^ Observed AIS cause rates suggested that adolescents and very young adults seemed closer together than very young adults and young adults could be. Their main stroke aetiologies were the same, namely cardiac disorders, arteriopathy and prothrombotic disorders, with no significant difference in stroke distribution either with the TOAST classification or the paediatric classification. Moreover, the paediatric classification seemed to describe better adolescents’ and very young adults’ stroke causes, with a smaller rate of cryptogenic stroke using this classification. Of note, adolescents and very young adults had a lower rate of intracranial arteriopathy than reported in paediatric studies, where it represents the main AIS cause category.^27–29^ However, there is still no definite evidence on how stroke in adolescents differs from those in younger children (eg, < 10 years) in terms of aetiology, recurrence rates and outcomes. The peak age during adolescence points to an expanded or age-modified risk profile, which needs to be investigated in larger prospective studies and data collections. For the future, a structured, stepwise approach to diagnostic work-up would be beneficial for improving clinical workflows and decision-making, being also more cost-effective. Such an approach can enhance the consistency and thoroughness of diagnostics, particularly when dealing with complex or unclear cases.

A remarkable finding in this analysis is the sex-related difference between age groups, with a predominance of males during adolescence and an increasing proportion of females transitioning into young adulthood. Explicit causes for this are not specified yet; endogenous and hormonal factors are discussed as well as usage of (illicit) drugs and alcohol.^30,31^ Female-specific risk factors in young adulthood, particularly the use of oral contraceptives and their specific composition, as well as pregnancy and the postpartum period, need to be evaluated both independently and in interaction with additional comorbidities and risk factors such as migraine, thrombophilia, PFO and autoimmune diseases. The aforementioned considerations should also be taken into account when interpreting the peak incidences observed in this analysis, occurring at ages 13–18 years (higher in males) and, to a lesser extent, at age 25 years (higher in females). Further studies are needed to identify specific correlations and to optimize potential primary or secondary preventive treatment strategies.

The high rate of cryptogenic stroke in both adolescents and very young adult groups raises issues about the accuracy of stroke cause inquiry. Beyond identified cause, the identification of risk factors is also important. Whereas a cause is a direct trigger or mechanism that leads to a development of a disease, a risk factor increases the likelihood of developing a specific disease without being directly causal.^32^ Considered to be relevant risk factors for AIS are prothrombotic disorders. However, they are emphasized to be conditions that are generally permissive rather than isolated causes. Hypercoagulable states were common, but often occurred alongside other risk factors, and clear patterns of isolated prothrombotic abnormalities were not observed.^33^ Therefore, particularly in the absence of a PFO, prothrombotic disorders are viewed as contributory rather than sufficient for stroke causation.

In this study, strokes associated with a PFO were classified according to the adult modified TOAST system. In cases where no other prothrombotic or etiological factors were identified, these events were assigned to the category of undetermined cause, as per the TOAST definition. Therefore, the high proportion of cryptogenic strokes observed in our cohort may in part reflect this classification approach rather than a true absence of identifiable aetiology. This emphasizes that PFO-related strokes and truly cryptogenic strokes are grouped together in the TOAST system, which should be considered when interpreting the frequency of cryptogenic events in young patients. In addition, PFO prevalence is markedly higher in younger stroke patients, and its precise role in stroke aetiology within this age group remains uncertain.^34,35^ These observations highlight the importance of a comprehensive evaluation in young stroke patients with a PFO to exclude other potential causes.

Furthermore, data from the French study suggested a considerable prevalence of dyslipidaemia (30%), smoking (17%) and overweight/obesity (17%) among adolescents with AIS.^12^ In the overall data of the ODYSSEY study including adults aged 18–49 years, notable prevalences for risk factors of early atherosclerosis are also highlighted with dyslipidaemia in 65.4% and smoking in 49.6%.^21^ With rising incidence of obesity starting in childhood, existing evidence indicates that primary prevention of atherosclerotic disease should begin in childhood.^36^ This comes along with previous data suggesting that dyslipidaemia and hypertriglyceridemia may be more prevalent in children with AIS compared with stroke-free children.^37^ The causality of these risk factors in young age still needs to be further evaluated, although it has been shown that the increasing incidence of AIS in young adults coexist with rising prevalence of cardiovascular risk factors.^38,39^ Further unified investigations in adolescents and very young adults ≤30 years with stroke are of great interest and could potentially lead to new therapeutic and educational approaches as this might be a potential modifiable vascular risk factor contributing to stroke recurrence and mortality. In this context, it is worth considering to what extent these vascular risk factors—primarily assumed in older adults—should be generally incorporated into risk factor classifications for strokes in children and especially adolescents.

The type of used classification may also influence the rate of cryptogenic stroke. In this study, we chose to compare the TOAST classification and the paediatric classification used in the German ESPED study,^9^ because recorded data in the three samples from different countries could fit in these classification. Another common classification system for paediatric stroke is the Childhood AIS Standardized Classification and Diagnostic Evaluation (CASCADE), developed in 2011.^40^ In this study, we chose not to use CASCADE as a paediatric classification system as it is primarily based on the anatomic structure whereas we wanted to focus on causes but also risk factor profile. The proportion of the category “other” with undetermined aetiology/aetiology unclear despite complete workup would probably have been relatively high—as it was the case in 45% in analysis of the French data also used in this analysis.^12^ Another option would have been analysing data using the International Pediatric Stroke Society (IPSS) classification system as it was used in the ODYSSEY study.^20,21,28^ But varying data collection especially in the group of adolescents due to different study designs would have made analysis inconsistent with numerous missing data.

Interestingly, AD rate was the only significant difference observed between adolescents and very young adult AIS cause, with an increased ratio in very young adults. As previously reported, AD seems to be more frequent on cervical locations in young adults and on intracranial locations in adolescents, with possible diagnostic overlap between intracranial dissection and focal cerebral arteriopathy.^41^ History of migraine, neck manipulation and hypertension are associated with cervical AD in young adults.^42,43^ To what extent possible factors such as hormonal changes, neck growth, genetic or other suspected factors influence increasing rate of cervical dissection over age remains to be investigated.

### Strengths and limitations

This study relies on a large number of cases of a rare disease in this age group, recorded during a similar time period in 3 north-western European countries with comparable sociodemographic backgrounds and similar healthcare systems. It thus allowed to draw interesting conclusions about the repartition of AIS causes found in this subpopulation. As data from three different cohort studies with different study designs were pooled, we could not avoid related consequent biases. Etiologic work-up harboured many similarities but was not conducted uniformly. Therefore, a key limitation of this study is the absence of standardized investigation protocols and that the completeness of etiological evaluations was not systematically captured. As a result, despite the aim for completeness, some risk factors may be underreported due to limited assessments, and the proportion of childhood strokes classified as undetermined origin may be overestimated. For example, 8.1% (Dutch ODYSSEY study) of the patients without a cause of stroke did not receive a cardiac ultra-sound, or transthoracic echocardiography was conducted without bubble study, potentially leading to overestimation of the number of cryptogenic strokes and an underestimation of the number of patent foramen ovale.^21^ Similarly, due to non-uniform data collection across the cohort studies, not all aspects could be examined that would be of interest in investigating and comparing the etiological and risk profiles in between the age groups, such as the use of oral contraceptives, the prevalence of elevated lipoprotein(a), or the distinction between migraine with and without aura.

## Conclusion

This study demonstrates that adolescents and very young adults with AIS share relevantly overlapping etiological patterns and risk factor profiles, suggesting that these patients may represent a common age group rather than two distinct cohorts. While certain differences were observed, similarities clearly outweighed differences. Sex-related differences in between age groups remain insufficiently understood and highlight the need for further studies to clarify their role in stroke aetiology in this age range.

The high proportion of cryptogenic strokes observed underscores the limitations of current adult-oriented classification systems in capturing the heterogeneity and transitional nature of stroke in adolescents and very young adults. These findings support the need for an age-adapted risk factor classification approach that integrates both paediatric-specific and early adult risk factors.

## Previous submission

The manuscript has not been submitted elsewhere nor published elsewhere in whole or in part, except as an abstract. Preliminary data were presented orally at the congress of the German-speaking Society for Neuropediatrics (GNP, Stuttgart, Germany, October 2024; Abstract: Neuropediatrics 2024; 55(S01):S1-S25; https://doi.org/10.1055/s-0044-1791891).
